# A Heme Oxygenase-1 Transducer Model of Degenerative and Developmental Brain Disorders

**DOI:** 10.3390/ijms16035400

**Published:** 2015-03-09

**Authors:** Hyman M. Schipper, Wei Song

**Affiliations:** 1 Bloomfield Centre for Research in Aging, Lady Davis Institute for Medical Research, Jewish General Hospital, Montreal, QC H3T1E2, Canada; E-Mail: wsong39@yahoo.com; 2 Department of Neurology & Neurosurgery and Department of Medicine, McGill University and Jewish General Hospital, Montreal, QC H3T1E2, Canada

**Keywords:** Alzheimer disease, astrocyte, carbon monoxide, heme oxygenase-1, iron, macroautophagy, mitochondria, oxidative stress, Parkinson disease, schizophrenia

## Abstract

Heme oxygenase-1 (HO-1) is a 32 kDa protein which catalyzes the breakdown of heme to free iron, carbon monoxide and biliverdin. The *Hmox1* promoter contains numerous consensus sequences that render the gene exquisitely sensitive to induction by diverse pro-oxidant and inflammatory stimuli. In “stressed” astroglia, HO-1 hyperactivity promotes mitochondrial iron sequestration and macroautophagy and may thereby contribute to the pathological iron deposition and bioenergetic failure documented in Alzheimer disease, Parkinson disease and certain neurodevelopmental conditions. Glial HO-1 expression may also impact neuroplasticity and cell survival by modulating brain sterol metabolism and the proteasomal degradation of neurotoxic proteins. The glial HO-1 response may represent a pivotal transducer of noxious environmental and endogenous stressors into patterns of neural damage and repair characteristic of many human degenerative and developmental CNS disorders.

## 1. Transduction and Pathogenesis of Chronic Human Brain Disease

Human neurodegenerative and neurodevelopmental disorders vary with respect to risk factors, sex predilections, ages of onset, regions of the neuraxis involved, behavioral and neurological symptoms, hallmark cellular inclusions (if any), neurochemical disturbances, structural and functional neuroimaging, and electrophysiology. The advent of successful therapeutic interventions to prevent, attenuate, arrest or reverse neuronal depletion and clinical decline in these conditions pre-supposes a thorough understanding of intrinsic central nervous system (CNS) responses to stressors and their relationship to cytopathological mechanisms that underlie clinical disease expression. Classically, specific pharmacotherapeutics and non-pharmacological interventions have been implemented to manage these conditions commensurate with the perceived etiopathogenesis or, when the latter is uncertain, the neurobehavioral phenotype (symptomatic treatment). Thiamine replacement in cases of Wernicke encephalopathy would be an example of the former; levo-dopa administration for the amelioration of extrapyramidal symptoms (tremor, rigidity, *etc.*) in Parkinson disease (PD) patients would exemplify the latter. A second approach, and the focus of our laboratory, is to delineate “core” neuropathological mechanisms operating in many, if not all, chronic CNS disorders and develop therapeutic strategies targeting these shared manifestations. While this approach may not directly address disease “etiology” (which may differ substantially among the diverse entities), it may disrupt salient common pathways that drive disease “pathogenesis” and thereby achieve meaningful clinical outcomes. In this article, we review evidence that (1) iron deposition, oxidative stress (OS), mitochondrial injury and macroautophagy constitute a single neuropathological “lesion” which may foster progression of various degenerative and developmental brain disorders and (2) these ubiquitous neuropathological changes result from the over-expression of astroglial heme oxygenase-1 (HO-1) in the affected neural tissues. We conclude by suggesting that glial *HMOX1* induction, while conferring certain neuroprotective benefits, may be a pivotal transducer of noxious ambient stimuli (risk factors) into patterns of aberrant brain structure and function in various human degenerative and developmental CNS disorders.

## 2. Convergent Neuropathology in Chronic CNS Disorders

On the basis of evidence adduced from human neuropathological surveys, animal models of CNS disease and cell culture experiments, we posited that *augmented iron deposition*, *oxidative stress*, *mitochondrial insufficiency* and *macroautophagy* which have been amply documented in numerous chronic brain disorders may be viewed as a single neurohistopathological “lesion”, with the advent of one component facilitating the appearance of the others [1]. This review will focus largely on Alzheimer disease (AD), Parkinson disease (PD) and schizophrenia (SCZ), with the understanding that many of the principles outlined may be germane to a host of other neurological and psychiatric illnesses.

### 2.1. Alzheimer Disease

AD is a late-onset dementing disorder featuring progressive neuronal degeneration, gliosis, and the accumulation of extracellular deposits of amyloid (senile plaques) and intracellular inclusions (neurofibrillary tangles) in the basal forebrain, hippocampus, and association cortices [2]. Oxidative stress and mitochondrial insufficiency in AD brain are evidenced by (i) a relative abundance of mtDNA deletions and mis-sense mutations [3,4] which correlate with levels of free radical damage [5] (ii) deficiencies in Kreb cycle enzymes and components of the electron transport chain [6]; (iii) altered mitochondrial morphology and turnover (mitophagy) in the affected tissues [7] and (iii) suppressed cerebral metabolism (glucose utilization) in positron emission tomography studies [8,9]. Likely sources of enhanced OS in the AD brain include production of reactive oxygen species (ROS) by senescent mitochondria, accelerated β-amyloid deposition [10], elaboration of tumor necrosis factor-α (TNFα), interleukin-1 β (IL-1β) and nitric oxide (NO) by activated microglia [11] and excessive sequestration of non-transferrin iron in the diseased neural tissues [12,13].

### 2.2. Parkinson Disease

Idiopathic PD is an aging-related movement disorder featuring progressive degeneration of dopaminergic (DA) neurons in the substantia nigra pars compacta, the genesis of intraneuronal fibrillar inclusions (Lewy bodies) and variable loss of serotonin and noradrenaline in other brain stem nuclei. As in the case of AD, there is considerable evidence of transferrin-independant iron trapping, oxidative substrate damage, bioenergetic failure and macroautophagy in the PD-affected brain tissues [12,14,15]. The pathological iron stores, accelerated DA turnover (generating H_2_O_2_, ortho-semiquinones), TH1 cytokines, NO, and MPTP-like xenobiotics may give rise to toxic pro-oxidants in the PD basal ganglia [16]. In PD-affected brain tissues, weakened mitochondria may represent both sources and chief targets of the excess ROS, as indicated by (i) diminished cytochrome subunit expression and Complex I activity [17]; (ii) oxidative mtDNA lesions in neurons and astroglia [18]; and (iii) suppressed glucose utilization and augmented lactate production *in vivo* [19].

### 2.3. Schizophrenia

SCZ is a developmental, neuropsychiatric condition of early adulthood characterized by psychosis, cognitive dysfunction, aberrant emotional displays, hyperkinetic/stereotypic movements and social withdrawal. Although subtle relative to the degenerative changes observed in AD and PD, the “core” neuropathological features of OS [20], altered brain iron distribution [21], mitochondrial insufficiency and macroautophagy [20,22] have been reported in the SCZ brain. In the following sections, we review evidence that the establishment of this core neuropathological tetrad may be contingent on the antecedent up-regulation of HO-1 in astrocytes.

## 3. Heme Oxygenase-1

### 3.1. HO-1 Regulation and Physiology

**Figure 1:**
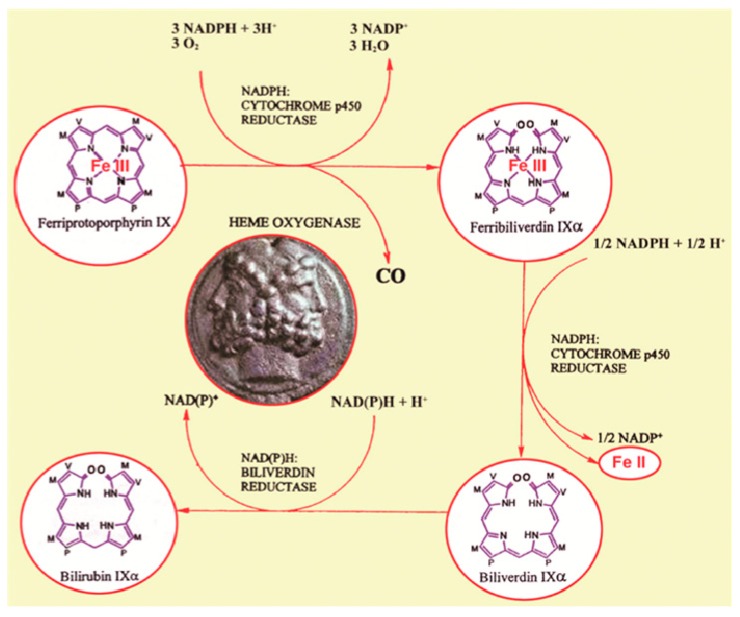
The heme catabolic pathway. The heme degradation products, ferrous iron (Fe II), carbon monoxide (CO), and biliverdin/bilirubin may behave as either pro-oxidants or antioxidants accounting for the disparate influences of heme oxygenase expression on cell function and survival (symbolized by Janus faces). M = methyl; V = vinyl; P = propionate (from [27], with permission).

**Figure 2:**
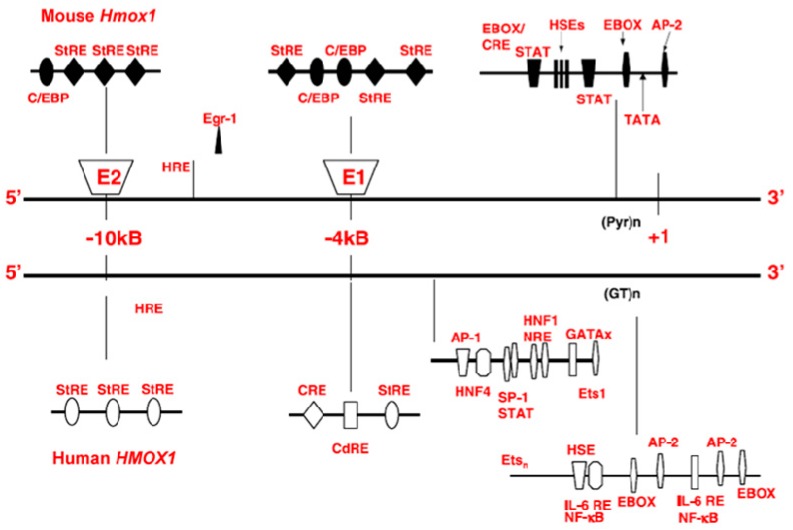
Regulatory domains of the mouse and human *Hmox1* genes. AP-1, activator protein 1; AP-2, activator protein-2; CdRE, cadmium responsive element; C/EBP, CAAT/enhancer binding protein binding site; CRE, cAMP responsive element; egr1, early growth response factor-1 binding site; ets, ets binding site; GATAx, GATA binding proteins; (GT)n, GT-rich repeat region; HNF1/4, hepatocyte nuclear factor 1/4; HRE, hypoxia responsive element; HSE, heat shock element; NF-κB, nuclear factor κ-B; NRE, negative regulatory element; (Pyr)n, pyrimidine-rich region; SP-1, specificity protein-1; STAT, signal transducer and activator of transcription; StRE, stress responsive element; TATA, TATA box; USF, upstream stimulatory factor. (Modified from [28], with permission).

Heme degradation in mammals is mediated by the heme oxygenase family of enzymes (E.C. 1:14:99:3; heme-hydrogen donor:oxygen oxidoreductase). These enzymes are mainly situated within the endoplasmic reticulum where they act, together with NADPH cytochrome P450 reductase, to oxidize heme to biliverdin, free ferrous iron and carbon monoxide (CO; Figure 1). Biliverdin is catabolized further to the bile pigment, bilirubin by biliverdin reductase (BVR) [23]. Mammalian tissues express two isoforms of heme oxygenase, HO-1 (a.k.a. HSP32) and HO-2. A third protein, HO-3 is considered a retrotransposition of the HO-2 gene unique to rats [24]. Basal HO-1 expression in the normal brain is confined to small groups of scattered neurons and neuroglia [25] whereas HO-2 protein is more broadly deployed throughout the neuraxis [26]. The *Hmox1* promoter (Figure 2) contains a remarkable array of response elements which render the gene exquisitely sensitive to induction by a wide range of inflammatory and pro-oxidant stimuli including heme, β-amyloid, DA, lipopolysaccharide, IL-1β, TNFα, cysteamine (CSH), heavy metals, H_2_O_2_, hyperoxia, and UV light. HO-1 expression is induced by Nrf2 transcription factor binding to Maf response elements (MARE) in the *Hmox1* promoter and repressed by the heme-regulated protein, Bach1 [27,28]. In virtually all cells, OS may transiently increase the intracellular “free heme pool” by promoting the denaturation of hemoproteins such as cytochromes, respiratory burst enzymes, myoglobin and peroxidases [23]. Under conditions of stress, the up-regulation of HO-1 may confer cytoprotection by accelerating the breakdown of pro-oxidant heme to the radical-scavenging bile pigments, biliverdin and bilirubin [29,30,31,32]. Under these conditions, apoferritin, a major ferroxidase and iron binding protein, is often co-induced to prevent potential toxicity accruing from the intracellular liberation of heme-derived ferrous iron [23,33]. As described below, heme-derived iron and CO may, under certain circumstances, *amplify* intracellular OS and substrate damage by stimulating the formation of ROS within the mitochondrial compartment [23,27,34,35].

### 3.2. HO-1 in Human Brain Aging and Disease

Numbers of neuroglia immunoreactive for HO-1 accumulate progressively with advancing aging in the normal human brain [36]. In senescent neural (and possibly other) tissues, HO-1 decorates and may be responsible for the biogenesis of corpora amylacea, glycoproteinaceous inclusions commonly encountered in aging mammalian cells [1,37]. The fraction of glial fibrillary acid protein (GFAP)-positive astrocytes that expresses HO-1 is markedly increased in the hippocampus and cerebral cortex of patients with AD relative to age-matched, non-demented controls. Enhanced up-regulation of glial HO-1 is already apparent in the brains of subjects with mild cognitive impairment (MCI), a frequent harbinger of incipient AD [27]. In the MCI temporal cortex, numbers of astrocytes immunoreactive for HO-1 correlated with the degree of neurofibrillary pathology and was associated with diminished performance in tests of global cognition, episodic memory, semantic memory and working memory. Astroglial HO-1 expression in the hippocampus was similarly associated with lower scores for global cognition, perceptual speed and semantic memory. The MCI data establish that glial *HMOX1* induction is a relatively early event in the pathogenesis of sporadic AD. Butterfield and colleagues concur with this conclusion but caution that measurement of HO-1 and BVR protein levels *per se* may not fully represent the brain’s capacity to degrade heme in MCI/AD. The Butterfield lab adduced evidence for diminished BVR *activity* in the MCI/AD brain which they attributed to excessive oxidative post-translational modification of the BVR protein [38,39]. If confirmed, this could be an important mechanism whereby insufficient bilirubin is generated to offset the neurotoxic effects of heme-derived iron/CO in this disease.

In PD, HO-1 co-localizes with Lewy bodies within affected dopaminergic perikarya and is highly over-expressed in astrocytes of the substantia nigra [40]. Regarding human neurodevelopmental disorders, Prabakaran *et al.* reported *HMOX1* gene induction associated with evidence of altered redox homeostasis and mitochondrial dysfunction in the prefrontal cortex of patients with SCZ [20]. Robust elaboration of HO-1 also occurs in the brains of patients with progressive supranuclear palsy, frontotemporal dementia, corticobasal ganglionic degeneration, multiple sclerosis, ischemic and hemorrhagic stroke, and cerebral malaria [41].

### 3.3. HO-1: Mediator of “Core” Neuropathology in Stressed Astroglia

**Figure 3:**
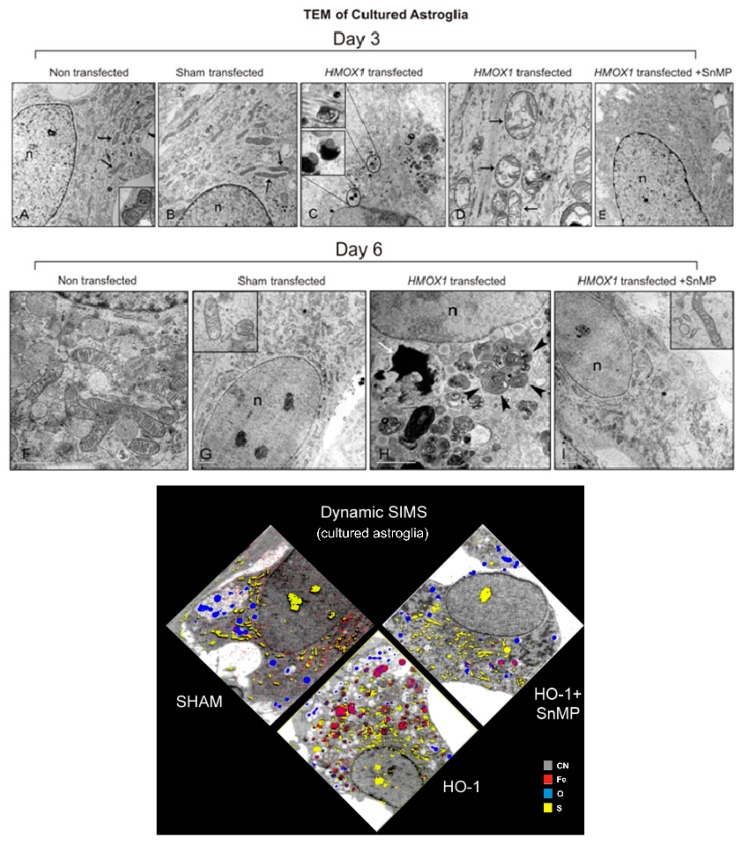
Effects of *HMOX1* transfection on astrocyte ultrastructure and iron deposition. *HMOX1*-transfected cells (**C**,**D**,**H**) contain degenerate mitochondria (straight black arrows), cytoplasmic autophagic and multilamellar bodies (**C** inset, arrowheads) and lipofuscin-like material (white arrow). These pathological profiles are not seen in non-transfected (**A**,**F**) and sham-transfected (**B**,**G**) cells or in *HMOX1*-transfected cells treated with the HO inhibitor, SnMP (**E**,**I**). (**J**) Dynamic secondary ion mass spectroscopy (SIMS) of sham-transfected astrocytes, *HMOX1*-transfected cells (HO-1) and *HMOX1*-transfected cells exposed to SnMP (HO-1 + SnMP). Ions of interest (colored) are superimposed on cytoarchitecture (grey-scale) visualized by CN mapping. Note robust iron deposition (red) in *HMOX1*-transfected cells, an effect that is abrogated by exposure to SnMP. Magnification bars = 2 μm in panels **A**–**C**,**E** & **G**–**I**, and 1 μm in panels **D** & **F**. SnMP = tin mesoporphyrin. (Modified from [49], with permission).

A large body of evidence garnered in our laboratory since 1990 indicates that chronic or repeated induction of *HMOX1* in mammalian astrocytes promotes a cascade of cytopathological changes which may partially explain the tetrad of oxidative stress, non-transferrin iron deposition, mitochondrial membrane damage and macroautophagy characteristic of numerous degenerative, developmental and inflammatory CNS disorders. Key features germane to the current discussion are summarized here. A host of ROS, proinflammatory cytokines (e.g., TNFα, IL-1β), dopamine, β-amyloid and cysteamine stimulate HO-1 expression in cultured rat astroglia [42,43,44]. In these cells, ferrous iron and CO liberated by HO-1-mediated degradation of heme amplifies intracellular oxidative stress as evidenced by the accumulation of protein carbonyls, 8-epiPGF2α, 8-OHdG, several synthetic OS reporter molecules and compensatory up-regulation of manganese superoxide dismutase (MnSOD) [35,45,46]. Exposure of cultured astrocytes to the aforementioned *Hmox1* inducers for 3–6 days promotes the sequestration of non-transferrin ^59^Fe (or ^55^Fe) within mitochondria without affecting flux of the metal into whole-cell and lysosomal compartments, as determined by subcellular fractionation/scintillation counting and X-ray microprobe analysis [43,47,48]. Of note, mitochondrial iron trapping under these conditions was not demonstrable when diferric transferrin served as the iron donor (*ibid*.). Furthermore, mitochondrial iron sequestration in HO-1-transfected glia occurred in the absence of discernible changes in transferrin receptor, ferritin and ferroportin expression, iron regulatory protein (IRP)1 activity, and IRP2 levels [49]. The latter observations bolster the validity of our *in vitro* model because they are consistent with evidence that the transferrin pathway, pivotal for iron mobilization in most mammalian tissues, plays little or no role in the pathological deposition of the metal in AD- and PD-affected neural tissues [27]. In astrocytes treated with DA, TNFα or IL-1β, inhibitors of the mitochondrial permeability transition pore (cyclosporin A, trifluoperazine) attenuated mitochondrial iron trapping. Intracellular OS accruing from HO-1 activity likely promotes pore opening [50,51] and transfer of cytosolic iron to the mitochondrial matrix [27] (Figure 3). Importantly, all of the effects of HO-1 inducers described above were fully recapitulated in cultured astroglia transiently-transfected with human *HMOX1* cDNA in the absence of exogenous stressors [27]. These transfection experiments were critical because the stressors employed to induce *HMOX1* could theoretically have exerted their biological effects in our model by mechanisms unrelated to HO-1 action.

In the stressor-exposed and *HMOX1*-transfected glia, the mitochondrial influx of redox-active iron incurs membrane damage, dissolution or rearrangement of cristae, and organellar distension [27,49]. Enhanced infidelity of electron transport within injured inner mitochondrial membranes promotes the excessive production of superoxide (and other ROS) which, in turn, may predispose to oxidative mtDNA mutations. The latter code for aberrant cytochrome components which may further accelerate ROS generation by the impaired electron transport chain, thereby establishing a self-perpetuating cycle of oxidative mitochondrial damage and bioenergetic failure [52]. The impaired mitochondria merge with lysosomal constituents in a complex macroautophagic process which occasionally culminates in the genesis of corpora amylacea (CA) [37,49,53]. CA are glycoproteinaceous, ubiquitinated cytoplasmic inclusions that accumulate in periventricular and subpial regions of the human brain in the course of normal aging, and to a greater extent in patients with hippocampal sclerosis, MCI, AD and other neurodegenerative conditions [37,54,55]. The formation of CA in the *HMOX1*-transfected glia further supports our contention that HO-1 overexpression accelerates cytopathological processes germane to human brain aging and disease. Another salient observation was that the biochemical and morphological changes documented in the *HMOX1*-transfected astrocytes were abrogated by exposure to tin mesoporphyrin (SnMP), a competitive inhibitor of HO activity. The latter indicates that the behavior of HO-1 in our model is canonical (enzymatic) in nature and not secondary to novel signalling and transcription factor (non-enzymatic) roles ascribed to HO-1 in other systems [56].

One might justifiably argue that the HO-1-mediated gliopathy should prove clinically (neurologically) significant only if it somehow impacts the function or viability of nearby neuronal populations. To this end, we demonstrated that neuron-like PC12 cells grown on a substratum of *HMOX1*-transfected (iron-laden) astrocytes exhibit far greater susceptibility to oxidative killing than PC12 cells co-cultured with sham-transfected (control) astroglia, effects preventable by co-incubation of the cultures with antioxidants, iron chelators and SnMP [57]. Using electron spin resonance spectroscopy, we showed that the glial mitochondrial iron in these cells behaves as a “pseudoperoxidase” which, in the presence of hydrogen peroxide, promotes the oxidation of dopamine and catecholestrogens to neurotoxic ortho-semiquinone radicals [58,59]. The glial iron also facilitates the non-enzymatic bioactivation of the pro-neurotoxin, MPTP to the dopaminergic neurotoxin, MPP+ in the face of monoamine oxidase blockade [60]. Various ROS, MPP+ and other neurotoxins generated within the astroglial compartment may be extruded to the extracellular space [60,61] and thereby promote injury and dysfunction of vulnerable *neuronal* constituents [27,59]. Glial HO-1 overexpression may also impact neuronal cytokinetics, survival andplasticity by modulating brain sterol/oxysterol metabolism [46,62,63] and the proteasomal degradation of α-synuclein and tau, proteins heavily implicated in the pathogenesis of PD and AD [64]. These additional biochemical effects downstream of HO-1 action are beyond the scope of the current review, although they serve to further underscore the salience of the heme catabolic pathway to matters of CNS health and disease.

### 3.4. The GFAP.HMOX1 Transgenic Mouse

**Figure 4:**
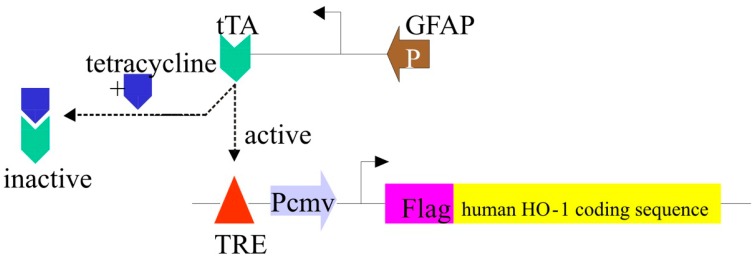
Design of conditional GFAP.HMOX1 transgenic mice. In absence of tetracycline/doxycycline, tTA binds to the tetracycline response element (TRE), thereby activating transcription of the Flag-tagged *HMOX1* gene under the minimal human cytomegalovirus (CMV) promoter/enhancer (Pcmv). The GFAP promoter restricts transgene expression to the astrocyte compartment. tTA is inactivated in the presence of doxycycline, thereby preventing expression of the *HMOX1* transgene. (From [65], with permission).

**Figure 5:**
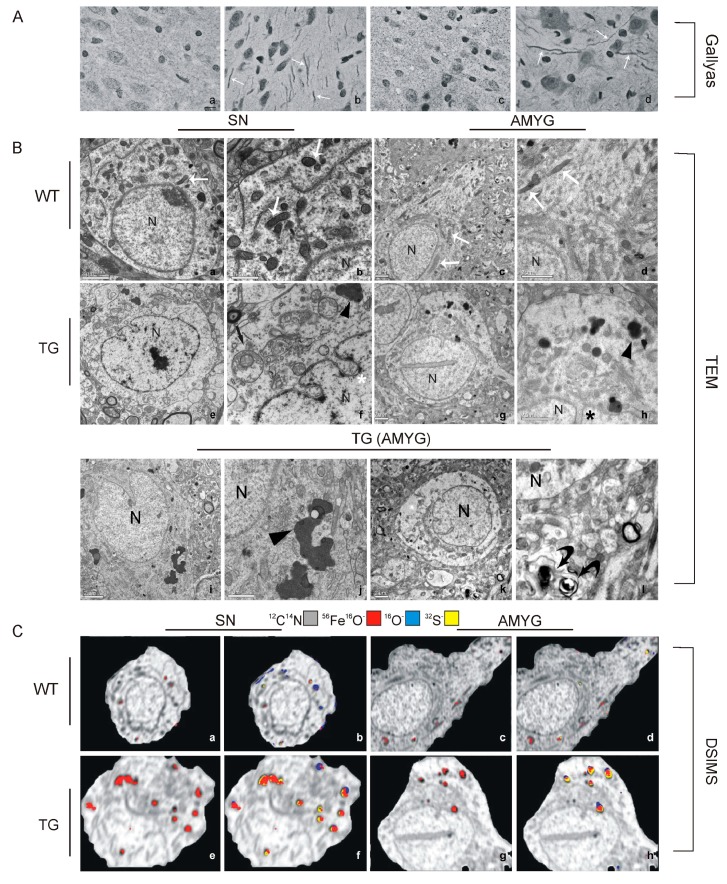
Neurohistopathology of 48 week-old GFAP.HMOX1 and wild-type (WT) mice. (**A**) Gallyas silver staining demonstrating abundant neuritic damage (arrows) in the GFAP.HMOX1 substantia nigra (SN; **a**) and amygdala (AMYG; **d**) but not in respective WT preparations (**a**,**c**). Magnification bar = 10 μm; (**B**) GFAP.HMOX1 and WT brain ultrastructure. Fields depicted in **a**, **c**, **e**, **g** are shown at higher magnifications in panels **b**, **d**, **f** and **h**, respectively. GFAP.HMOX1 astrocytes contain degenerate mitochondria (straight black arrow in **f**), autophagic and lipofuscin-like material (arrowheads), and nuclear envelope invaginations (asterisks). These pathological features were not seen in WT preparations. Degenerate neurites (curved arrows in l) were observed in close proximity to pathological astrocytes in TG, but not WT, brains; (**C**) Dynamic secondary ion mass spectroscopy (SIMS) of TG and WT astroglia from substantia nigra (SN) and amygdala (AMYG). Panels **a**, **c**, **e** and **g** depict FeO, and panels **b**, **d**, **f** and **h** depict multiple elements of interest, superimposed on cytoarchitecture (gray-scale) visualized by CN mapping. Little to no iron is detectable in WT preparations. Astrocytes in TG substantia nigra and amygdala, but not in respective WT preparations, are replete with iron-laden intracellular inclusions that co-localize with oxygen and sulphur. (Panels **A** and **B** from [65]; Panel **C** from [66], with permission).

To explore further the role of glial HO-1 in the development of the neuropathological “core”, we generated novel GFAP.HMOX1 transgenic mice that selectively and conditionally express the human *HMOX1* gene in astrocytes. The mice were bred on an FVB background and engineered to express GFAP.tTA.TRE.Flag.HMOX1 final constructs. Incorporation of the GFAP promoter selectively targets human *HMOX1* gene expression to the *astrocytic* compartment. A tetracycline-suppressible (“Tet-off”) promoter element and dietary doxycycline (DOX) administration allow for temporal control of transgene expression. Addition of the Flag sequence permits differentiation of the transgenic product from endogenous (mouse) HO-1 (Figure 4) [65]. After 48 weeks of continuous *HMOX1* induction, the mice exhibited the tetrad of cytopathology observed in *HMOX1*-transfected glial cultures, *viz*. brain iron deposition, OS, mitochondrial membrane damage and macroautophagy/corpora amylacea formation [37,65,66] (Figure 5). As in the case of the *HMOX1*-transfected astrocyte cultures [49], iron deposition in the brains of GFAP.HMOX1 mice appeared “unregulated” (transferrin pathway-independent) in so far as brain homogenates revealed no overt alterations in transferrin receptor, ferritin, ferroportin, IRP1 or IRP2 protein concentrations [66]. The augmented structural and molecular markers of macroautophagy documented in the GFAP.HMOX1 mouse brain [65] and *HMOX1*-transfected glial monolayers [49] may reflect a primary defect in the autophagy machinery resulting in the accumulation of degenerate mitochondria, autophagosomes and other cellular debris. Alternatively, macroautophagy under these conditions may be intrinsically normal (and even physiologically enhanced) but incapable of coping with the massive flux of degenerate organelles presented to the autophagy machinery for disposal. Further experimentation will be necessary to distinguish between these possibilities.

**Figure 6:**
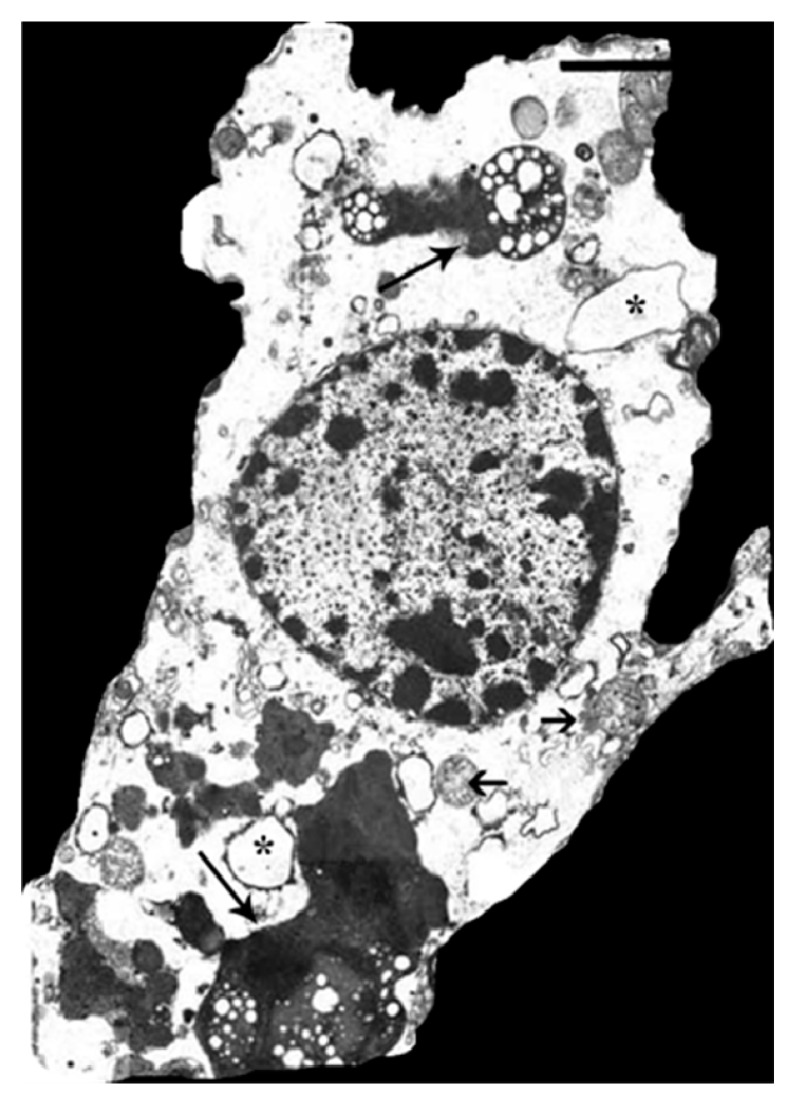
Hippocampal astrocyte pathology in human schizophrenia. Note the presence of swollen mitochondria (short arrows), distended cytoplasmic vacuoles (asterisks) and abundant autophagic material/lipofuscin (long arrows). Bar = 2 µm. Compare with cytopathology of *HMOX1*-transfected rat astroglia (Figure 3) and astrocytes of GFAP.HMOX1 transgenic mice (Figure 5). (From [22], with permission).

The gliodystrophic changes characteristic of the GFAP.HMOX1 mouse are highly reminiscent of ultrastructural abnormalities reported in the human SCZ hippocampus [22] (Figure 6). Moreover, the gliopathy in these mice is associated with subcortical neuritic degeneration (Figure 5) and an array of behavioral, neuroarchitectonic and neurochemical abnormalities reported in human SCZ and animal models of the disease: hyperlocomotion, behavioral stereotypy and impaired prepulse inhibition to acoustic startle; dysgenesis of the hippocampal dentate gyrus; increased basal ganglia DA and serotonin concentrations; and suppressed neuronal reelin immunoreactivity [65]. The advent of a SCZ-like phenotype in our animals is intriguing, as many (if not all) of the suspected “triggers” (risk factors) of SCZ, [67,68,69] are known inducers of *Hmox1* [27,33]. The gliopathological “core” lesion was also seen in older GFAP.HMOX1 mice which were manipulated to express the transgene between 8.5 and 19 months of age. However, rather than simulating SCZ, the neurophenotype of this “aging” model featured impaired motor coordination (rotarod test) and nigrostriatal *hypo*dopaminergia consistent with parkinsonism [70].

### 3.5. HO-1 Suppression in APP_swe_/PS1_ΔE9_ Transgenic Mice

Our observations *in vitro* and in the GFAP.HMOX1 mice suggest that up-regulation of HO-1 in astroglia not only facilitates the establishment of “generic” neuropathological lesions but that the latter may be causally linked to the clinical expression of various human neurodevelopmental and neurodegenerative disorders. To investigate this concept further, we ascertained the effects of pharmacological HO-1 suppression in adult APP_swe_/PS1_ΔE9_ transgenic mice, a well-characterized model of familial AD. OB-28 is an azole-based, brain-permeable inhibitor of HO-1. Unlike the metalloporphyrins (e.g., SnMP, ZnPPIX, *etc.*), which are relatively equipotent in their competitive inhibition of HO-1 and HO-2, OB-28 is fairly selective for the HO-1 isozyme. Daily intraperitoneal injections of APP_swe_/PS1_ΔE9_ transgenic mice with OB-28 (15 mg/kg) from 3 to 10 months of age significantly attenuated HO-1 activity in hippocampus and cerebral cortex with no overt untoward effects. Relative to saline-treated controls, animals receiving OB-28 exhibited reduced neuroinflammation (as measured by astroglial activation) without any change in the extent of amyloid deposition. Moreover, the OB-28-treated APP_swe_/PS1_ΔE9_ mice performed significantly better in a complex maze learning task compared to the controls [71]. These findings provide first proof-of-principle that targeted suppression of HO-1 activity may alleviate neuroinflammatory responses and behavioral deficits in a mouse model of AD independently of brain amyloid burden. Interestingly, Hui *et al.* [72] had previously shown that hyper-phosphorylation of tau, a key process in the pathogenesis of AD, is significantly enhanced in the brains of mice over-expressing HO-1 under control of the β-actin promoter, and that the former is likely iron-dependent. It remains to be seen whether OB-28 or other HO inhibitors suppress tau phosphorylation in animal models of AD.

## 4. An “HO-1 Transducer” Model of Chronic Brain Disease

**Figure 7:**
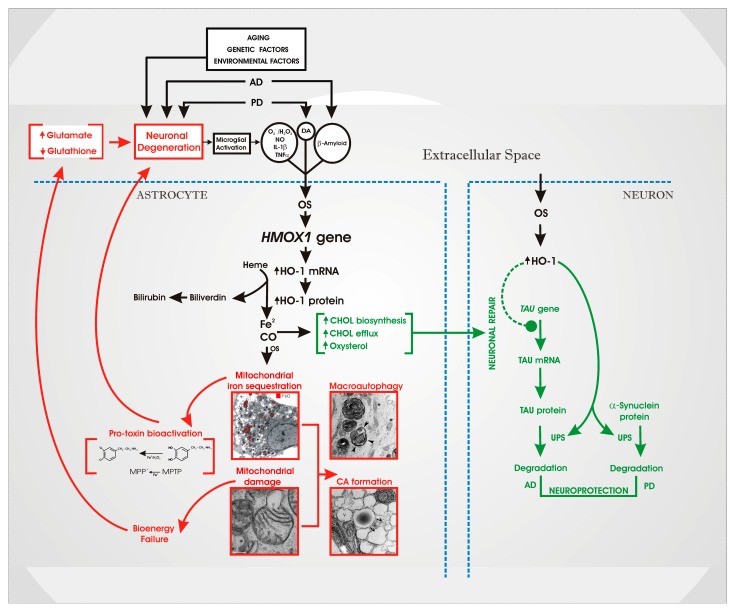
Putative roles of astroglial HO-1 in chronic human CNS disorders. Implications for AD and PD are emphasized. See text for details. AD, Alzheimer disease; CO, carbon monoxide; DA, dopamine; Fe^2^, ferrous iron; GSH, glutathione; HO-1, heme oxygenase-1; IL-1β, interleukin-1b; MPP+, methyl-4-phenylpyridinium; MPTP, 1-methyl-4-phenyl-1,2,3,6-tetrahydropyridine; NO, nitric oxide; OS, oxidative stress; PD, Parkinson disease; TNFα, tumor necrosis factor-α. (Modified from [27], with permission).

The data reviewed herein indicate that astroglial HO-1 is well-positioned to transduce a host of noxious stimuli and risk factors into “core” neuropathology common to many chronic CNS disorders, both developmental and degenerative. As schematized in Figure 7, sustained or repeated induction of *HMOX1* may amplify certain degenerative processes (depicted in red) in astrocytes while concomitantly activating several neuroprotective responses (green), a situation possibly reflecting *antagonistic pleiotropy* [73,74]. The dystrophic effects of HO-1 manifest as oxidative mitochondrial damage and ferrous iron sequestration which may, in turn, foster bioenergetic failure, macroautophagy and corpora amylacea formation, bioactivation of pro-neurotoxins, glutathione depletion (oxidative stress) and impaired clearance of synaptic glutamate (excitotoxicity). The ensuing neuronal injury may stimulate microglia to release proinflammatory cytokines (TNFα, IL-1β), ROS and nitric oxide (NO). The latter would further induce *HMOX1* in indigent astrocytes, completing a self-sustaining loop of pathological cellular interactions that may perpetuate oxidative damage and mitochondrial insufficiency within senescent and diseased neural tissues after exposure to initiating stimuli may have dissipated. Environmental and genetic risk factors may confer disease specificity by superimposing unique pathological signatures on this core lesion [27]. Furthermore, perinatal stressors activating this glial HO-1 cascade *prior to* the maturation of nigrostriatal and mesolimbic pathways may result in “hypertrophy” of dopaminergic projections and a neurodevelopmental hyperkinetic disorder (e.g., schizophrenia), whereas exposure of *established* dopaminergic circuitry to homologous influences in later life may yield neurophenotypes that are purely degenerative in nature (e.g., PD) [65].

## 5. Therapeutic Implications

Contemporary pharmacotherapy for PD, AD and SCZ is almost exclusively symptomatic in nature and effective neuroprotection would be a welcome development. The data reviewed here underscores astroglial HO-1 as a pivotal transducer of noxious stimuli, a potent driver of relevant cytopathology and a potential therapeutic target in these and other chronic human CNS disorders. Several metalloporphyrin inhibitors of heme oxygenase activity have found clinical use in the control of neonatal hyperbilirubinemia (jaundice) and certain adult liver conditions [75,76] and, if warranted, could be adapted for the treatment of neurodevelopmental and neurodegenerative conditions. Small, azole-based inhibitors, such as OB-28, may offer additional advantages for human use in light of their blood-brain barrier permeability, specificity for HO-1 relative to HO-2, and favorable toxicity profile in pre-clinical studies [71,77]. Alternatively, and spurred by successful deployment in hepatocytes [78] and neurons [79], siRNA delivery could be exploited to achieve selective HO-1 knock-down within the astrocytic compartment [80].

The “transducer” model presented here has emphasized the inimical face of neural HO-1 in chronic CNS disorders and largely reflects the contributions and perspective of the authors’ laboratory. However, there exists considerable evidence supporting beneficial roles for HO-1 in brain which cannot be ignored in the design and interpretation of human therapeutic trials. Evidence of HO-1-mediated neuroprotection has been adduced both *in vitro* and in animal models. For example, relative cytoprotection has been reported in neuroblastoma cells transfected with *Hmox1* cDNA following exposure to H_2_O_2_ [81,82] or β-amyloid_1-40_ [81]. Similarly, cerebellar granule cells cultured from transgenic mice designed to over-express HO-1 in neurons [83] appear to be relatively resistant to H_2_O_2_- and glutamate-mediated oxidative damage [84]. Moreover, HO-1 transgenic mice subjected to cerebral ischemia [85], brain or spinal cord trauma [86,87], or excitotoxin exposure [88,89] exhibit smaller infarct sizes and lower biochemical indices of neural injury. To a significant extent, the literature suggests that *Hmox1* induction is cytoprotective in *acute* models of CNS disease and injury (e.g., stroke), whereas *chronic* over-expression of neural HO-1 (as in the developmental and degenerative disorders) is predominantly dystrophic in nature. Yet, even in progressive neurodegenerative conditions, stressor-related activation of neural HO-1 may confer some degree of neuroprotection by inhibiting tau biosynthesis [82] or stimulating neuronal α-synuclein and tau degradation by the ubiquitin-proteasome system (Figure 7). The latter may serve to limit neurotoxicity in AD and PD accruing from the accumulation of protein aggregates [64]. To complicate matters further, glial *HMOX1* induction modulates sterol/oxysterol metabolism (Figure 7) in ways which may favour neuronal plasticity in the normal aging brain, but amplify neurodegenerative processes in conditions like AD [62,63,90]. Species and cell type differences, the temporal pattern and intensity of HO-1 expression, the chemistry of the local redox microenvironment, the diseases under consideration, and various experimental parameters may determine whether free radical damage resulting from the intracellular release of iron/CO or the antioxidant benefits of a suppressed heme:bilirubin ratio predominate. Further studies using well-defined disease models should help resolve many of these conflicting issues. Ultimately, human clinical trials may prove necessary to determine whether abrogation of the glial HO-1 response to noxious stimuli at strategic points of the life course will attenuate or exacerbate developmental and/or degenerative brain disease in high-risk populations.
